# The pursuit of novel head and neck cancer biomarkers – tissue and blood expression of chloride intracellular channels family

**DOI:** 10.1371/journal.pone.0333487

**Published:** 2025-10-24

**Authors:** Bartosz Paweł Wojtera, Kamila Ostrowska, Julia Ostapowicz, Mateusz Szewczyk, Julia Kozikowska, Wiktoria Maria Suchorska, Wojciech Golusiński

**Affiliations:** 1 Chair and Clinic of Head and Neck Surgery, Poznań University of Medical Sciences, Poznań, Poland; 2 Department of Head and Neck Surgery, Greater Poland Cancer Centre, Poznań, Poland; 3 Radiobiology Laboratory, Greater Poland Cancer Centre, Poznań, Poland; 4 Doctoral School, Poznań University of Medical Sciences, Poznań, Poland; 5 Department of Electroradiology, Poznań University of Medical Sciences, Poznań, Poland; University of the Republic Uruguay: Universidad de la Republica Uruguay, URUGUAY

## Abstract

**Introduction:**

The chloride intracellular channels (CLICs) engage in cancer pathogenesis and have been considered various cancer biomarkers and therapeutic targets. Preliminary research suggests CLICs may be important players in head and neck squamous cell carcinoma (HNSCC). There is a need for reliable HNSCC biomarkers besides well-known HPV and PD-L1.

**Aim:**

The study aimed to assess the role of CLICs in HNSCC pathogenesis and as potential disease biomarkers.

**Methods:**

We compared the *CLIC1–CLIC6* genes expression between the HNSCC tumors (n = 99) and the tissue from the free surgical margin (n = 74) at the mRNA level with RT-qPCR and at the protein level with Western Blot. To investigate the role of CLIC1-CLIC6 proteins as potential HNSCC blood biomarkers, we performed the ELISA assays on blood serum from 38 HNSCC patients and eight healthy individuals.

**Results:**

We found significant differences in the expression of every analyzed CLIC. At the mRNA level, *CLIC1* and *CLIC4* were overexpressed in oral cancer tissue, *CLIC3, CLIC5,* and *CLIC6* were down-expressed; in laryngeal cancer tissue, *CLIC2* and *CLIC3* were down-expressed. Tumor staging impacted *CLIC1* and *CLIC6* tissue expression, and histological grade impacted *CLIC6* tissue expression. At the protein level, CLIC3 was down-expressed in oral cancer tissue. Furthermore, CLIC1 and CLIC3 proteins were overexpressed, and CLIC4 and CLIC6 were down-expressed in the oral cancer patients’ blood serum compared to the control group.

**Conclusion:**

The different expression patterns of CLICs in HNSCC patients’ tissues and blood serum suggest that they may play an essential role in HNSCC pathogenesis and serve as biomarkers for HNSCC detection.

## Introduction

Head and neck cancer (HNC) arises in the upper parts of the digestive and respiratory systems, potentially impairing both functional and aesthetic aspects of this vital anatomical region [1]. In 2022, global incidence and mortality of HNC reached nearly 1,000,000 and 500,000 cases, respectively, making it the sixth most common type of cancer [2]. Head and neck squamous cell carcinoma (HNSCC) is the predominant variant of HNC. Well-known risk factors for HNSCC include tobacco smoking, alcohol consumption, and human papillomavirus (HPV) infection [3]. HNSCC treatment modalities include surgical resection, radiotherapy, chemotherapy, immunotherapy, and various strategies tailored to the individual patient following multidisciplinary evaluation [4].

Cancer biomarkers comprise diagnostic, disease-specific factors enabling early and specific diagnosis; prognostic factors stratifying patient prognosis (including overall survival and relapse-free survival), and predictive factors anticipating response to adequate therapeutic strategies [5]. Despite many proposed HNSCC potential biomarkers, only a few are used in everyday practice. In the 21st century, HPV infection gained interest as a prognostic and potentially diagnostic HNSCC biomarker, including p16 and HPV-DNA tumor expression and detection of circulating HPV-DNA in the blood [6–8]. However, HPV-related HNSCC is mainly associated with oropharyngeal cancer and plays a marginal role in the pathogenesis of HNSCC at other sites (3.9% of oral cancer and 2.8% of laryngeal cancer), where prevalence remains higher [2,9]. Another essential factor, PD-L1 expression, is an HNSCC prognostic and predictive biomarker in immune checkpoint inhibitor therapies [10]. PD-L1 is the only commonly used HNC biomarker listed by the US National Cancer Institute (https://www.cancer.gov/about-cancer/diagnosis-staging/diagnosis/tumor-markers-list, accessed on 19th October 2025). The limited number of validated HNSCC biomarkers emphasizes the necessity for further research.

Potential novel HNSCC biomarkers may be discovered among the chloride intracellular channels (CLICs)—a family of ion channels comprising six members: CLIC1, CLIC2, CLIC3, CLIC4, CLIC5, and CLIC6 [11]. Within cells, CLICs localize in membranes of organelles and plasmatic membranes and exist in soluble forms in the cytoplasm, engaging in multiple molecular pathways. CLICs participate in physiological and pathophysiological processes, including cardiovascular, respiratory, and nervous systems, hearing impairment, and cancer [12]. CLICs may also be secreted to the extracellular space and blood, which may challenge or complement their primary ion channel function [13–16].

Individual members of the CLIC family have been investigated across various types of cancer [12,17,18], although the family as a whole has only been analyzed in pancreatic ductal adenocarcinoma and hepatocellular carcinoma (HCC) through bioinformatic approaches [19,20]. The primary roles of CLIC proteins in cancer pathogenesis are diverse and member-specific. CLIC1 is implicated in multiple signaling pathways that drive proliferation, invasion, migration, and metastasis in a range of tumor types [18]. CLIC2 contributes to the formation and maintenance of tight junctions, suppresses the regulation of matrix metalloproteinase (MMP) activity, and inhibits malignant cell invasion and distant metastasis [13,21]. CLIC3 is involved in regulating cell cycle, focal adhesion, extracellular matrix interactions, and the p53 signaling pathway in bladder cancer [22]; it also facilitates late endosomal trafficking, contributing to tumor invasion across various cancer types [23]. CLIC4 plays context-dependent roles in tumor biology, acting as either a tumor suppressor or promoter by influencing cell proliferation, invasion, epithelial–mesenchymal transition (EMT), and tumor–stroma interactions [24]. CLIC5 contributes to cellular migration and invasion in HCC [25], inhibits lysosome-mediated apoptosis, and serves as a structural component of the actin cytoskeleton in placental microvilli [26]**.** CLIC6 is involved in ion transport regulation [27], mediates cell signaling pathways in HCC, and interacts with the tumor microenvironment (TME) in both HCC and breast cancer [28,29].

CLIC family members were previously considered potential cancer biomarkers and therapeutic targets in personalized medicine [17,30]. Several clinical studies have focused on the CLIC family in oncology, investigating tumor tissues, blood, and interstitial fluid expression, with the most interest in CLIC1 and CLIC4 [17]*,* whose expression may be associated with cancer progression [14,26,31,32], metastases [13,33,34], and radio- and chemoresistance [35–37].

Only a few studies have analyzed the importance of CLIC family members in HNC [31–33,38–46]. CLIC1 is essential in oral squamous cell carcinoma (OSCC), promoting OSCC progression by activating MAPK/ERK and MAPK/p38 pathways [31]. CLIC1 protein overexpression was found in the OSCC tissues [38,40] and in the blood of nasopharyngeal cancer (NPC) and OSCC patients [38,39]. Plasmatic CLIC1 expression may be associated with regional OSCC metastases [33]. CLIC4 protein overexpression was also observed in OSCC tissues, and its knockdown led to apoptosis via mitochondrial and endoplasmatic reticulum pathways [41]. CLIC4 is regulated by miR-142-3p, which is also overexpressed in OSCC tissues [42,47]. CLIC4 protein distribution within cells varies between cancerous and non-cancerous tissue, and the progression of lip HNSCC is linked to a shift in the CLIC4 expression pattern from nuclear to cytoplasmic localization [32].

There is a knowledge gap regarding CLIC2, CLIC3, CLIC5, and CLIC6 expression in HNSCC. Furthermore, no original study has investigated all CLIC family members in clinical material for any cancer. This study aimed to assess the role of the CLIC family members as potential HNSCC biomarkers. We analyzed *CLIC1–CLIC6* gene expression at the mRNA and protein level in HNSCC tumors and CLIC1*–*CLIC6 proteins expression in the blood serum of HNSCC patients. The novelty of our study includes the analysis of all CLIC family members in diverse tissues derived from HNSCC patients.

## Materials and methods

### Clinical material

Ninety-nine tumor tissues, 74 tissues harvested from the surgical margin (paired with the tumor tissues), and 38 blood samples were collected from patients during surgical resection in the Department of Head and Neck Surgery, Greater Poland Cancer Centre, Poznań, Poland, between 30^^th^^ May 2022 and 7^^th^^ August 2024. Samples were immediately snap-frozen in liquid nitrogen and stored at −80°C until RNA and protein isolation. Additionally, eight blood samples were collected from healthy volunteers on 6^^th^^ April 2024. The characterization of the total study cohort is presented in Table 1. For non-cancerous samples, the surgeon (B.W., M.S., W.G.) harvested the tissue adjacent to the surgical niche following tumor excision, but only if the corresponding surgical margin was > 0.5 cm in the intraoperative frozen section examination. Further, final histological evaluation was performed prior to analysis of the samples to rule out microscopic disease. In this article, the term ‘normal tissue’ refers to tissue harvested from the free surgical margin as described above.

**Table 1:** Patients and control group characteristics.

Tumor tissue mRNA expression group		Tumor tissue protein expression group		Blood serum protein expression group	
**Total tumor number**	**99**	**Total tumor number**	**69**	**Cancer patients**	**38**
**Total normal tissue number**	**74**	**Total normal tissue number**	**69**	**Healthy individuals**	**8**
**Tumor localization**		**Tumor localization**		**Tumor localization**	
Oral cavity	60	Oral cavity	44	Oral cavity	38
Floor of the mouth	25	Floor of the mouth	17	Floor of the mouth	13
Tongue	22	Tongue	20	Tongue	18
Floor of the mouth and tongue	9	Floor of the mouth and tongue	6	Floor of the mouth and tongue	6
Lower lip mucosa	2	Lower lip mucosa	1		
Buccal mucosa	1			Buccal mucosa	1
Gingival mucosa	1				
Larynx	39	Larynx	25		
**Age**		**Age**		**Age**	
Mean (±SD)	63 ± 10	Mean (±SD)	63 ± 11	Mean (±SD)	61 ± 9
Median	63	Median	64	Median	61
Range	26-90	Range	39-85	Range	39-84
**Sex**		**Sex**		**Sex**	
Male	73	Male	49	Male	27
Female	26	Female	20	Female	11
**pT stage**		**pT stage**		**pT stage**	
T1	5	T1	4		
T2	23	T2	16	T2	11
T3	29	T3	19	T3	16
T4	42	T4	30	T4	11
**pN stage**		**pN stage**		**pN stage**	
N0	41	N0	29	N0	10
N1	21	N1	12	N1	9
N2	27	N2	19	N3	10
N3	10	N3	9	N3	9
**Histological grade**		**Histological grade**		**Histological grade**	
G1	17	G1	11	G1	6
G2	65	G2	47	G2	25
G3	17	G3	11	G3	7
**Margin status**		**Margin status**		**Healthy individuals**	
R0	74	R0	69	**Age**	
Close (1–5 mm)	16	Close (1–5 mm)	–	Mean (±SD)	62 ± 9
R1	9	R1	–	Median	58
				Range	53-74
				**Sex**	
				Male	5
				Female	3

The inclusion criteria included adult patients of both sexes with HPV-negative, histologically confirmed, primary squamous cell carcinoma of the oral cavity and larynx, of all stages of the tumor and the regional metastases. Eligible patients were qualified for primary surgical treatment by a multidisciplinary tumor board. The exclusion criteria included synchronous tumors, recurrent tumors, other past primary tumors, and distant metastases. All participants signed informed written consent to participate in the study. The procedures were approved by Poznań University of Medical Sciences Bioethics Commission number 386/22 and were conducted with the principals of the Declaration of Helsinki.

### RNA isolation, reverse transcription, RT-qPCR analysis

The expression of *CLIC1-CLIC6* genes at the mRNA level in tumor and normal tissues was analyzed using the quantitative Real-Time Polymerase Chain Reaction (RT-qPCR). Total RNA was isolated from tissues using the RNeasy Mini Kit (Qiagen, Hilden, Germany). RNA samples were quantified by spectrophotometry and assessed for quality by gel electrophoresis. Subsequently, the samples were reverse-transcribed into cDNA using the RevertAid First Strand cDNA Synthesis Kit (Thermo Fisher, Waltham, MA, USA), with 500 ng of total RNA. RT-qPCR was performed using the CFX96 Real-Time System (Bio-Rad, Hercules, CA, USA) with Power Track SYBR Green Master Mix (Thermo Fisher, Waltham, MA, USA). The gene expression levels were normalized to the GAPDH housekeeping gene, and the relative expression levels were determined by the Pfaffl method. The list of primers is available in S1 File Supporting Information.

### Western blot analysis

The protein expression of CLIC1, CLIC3, and CLIC4 was analyzed using Western blot. Protein isolation was performed in RIPA buffer with protein inhibitors. The samples were separated by SDS-PAGE to quantify the selected protein level using Mini-PROTEANTGX precast gels (Bio-Rad, Hercules, CA, USA). Subsequently, the gel was transferred to a PVDF membrane using Trans-Blot Turbo transfer packs (Bio-Rad, Hercules, CA, USA) and blocked with 5% milk in TBST buffer. Immunodetection of bands was performed with rabbit polyclonal (Rp) anti-CLIC1 (Thermo Fisher Scientific Cat# PA5–41004, RRID: AB_2610527, Waltham, MA, USA), Rp anti-CLIC3 (Thermo Fisher Scientific Cat# PA5–82228, RRID: AB_2789389, Waltham, MA, USA), and Rp anti-CLIC4 (Thermo Fisher Scientific Cat# PA5–82328, RRID: AB_2789487, Waltham, MA, USA), followed by incubation with polyclonal goat anti-rabbit HRP-conjugated Ab (Thermo Fisher Scientific Cat# 31460, RRID: AB_228341). Rp anti-beta-tubulin Ab (Thermo Fisher Scientific Cat# PA5–16863, RRID: AB_10986058, Waltham, MA, USA) was used as the reference protein. Bands were visualized using Clarity Western ECL Blotting Substrate (Bio-Rad, Hercules, CA, USA) and the ChemiDoc™ Touch Imaging System (Bio-Rad, Hercules, CA, USA).

Antibody optimization was performed for CLIC1, CLIC3, and CLIC4 by testing various dilutions of the primary antibody (1:500 and 1:1000) and the secondary antibody (1:15,000, 1:10,000, 1:5,000, 1:2,500). The final dilutions were selected based on the optimal visualization signal: CLIC1–1:1000 (primary), 1:5000 (secondary); CLIC3–1:500 (primary), 1:5000 (secondary); CLIC4–1:1000 (primary), 1:5000 (secondary). Anti-beta-tubulin antibody was optimized in previous experiments, with a final dilution of 1:2500 (primary) and 1:10,000 (secondary).

### ELISA

To quantify CLIC1-CLIC6 protein levels in serum samples, specific AffiELISA Human chloride intracellular channel protein ELISA kits (CLIC1-CLIC6) (AffiGEN, Baileys Harbor, WI, USA) were used according to the manufacturer’s protocol. Briefly, assay samples and buffer were incubated with CLIC-HRP conjugate in a pre-coated plate for one hour. The wells were decanted, washed, and incubated with an HRP enzyme substrate and stop solution. The color intensity was measured spectrophotometrically at 450nm using a microplate reader. A standard curve was used to determine the concentration of CLIC1-CLIC6 in the serum samples.

### Statistical analysis

Statistical analyses were performed using GraphPad Prism v.8.1.1 (GraphPad Software, Inc., La Jolla, CA, USA). P values < 0.05 were considered statistically significant. All analyses were preceded by outlier identification using the Robust Regression and Outlier Removal (ROUT) method with a maximum false discovery rate (Q) set to 1%, followed by the exclusion of detected outliers. Afterward, a normality test was performed using the Shapiro-Wilk test. Depending on normality, comparisons between the two groups were conducted using either the unpaired two-tailed t-test or the two-tailed Mann-Whitney U test; for comparisons involving more than two groups, ANOVA or the Kruskal-Wallis test was used, followed by multiple comparison tests (Tukey or Dunn). Correlations were assessed using either the Pearson or Spearman correlation test, depending on data normality.

## Results

### Expression of CLICs differs between HNSCC and adjacent normal tissue

**Fig 1:**
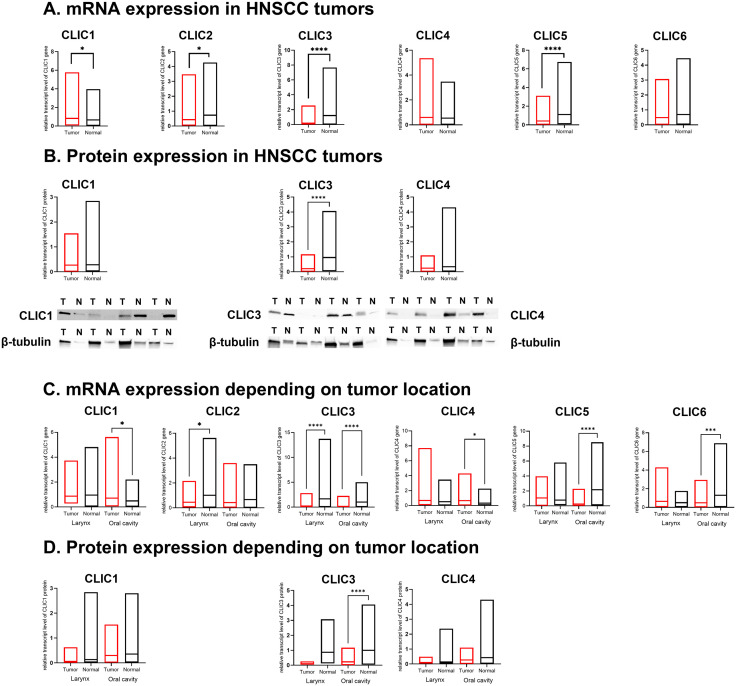
Chloride intracellular channels genes expression in the head and neck squamous cell carcinoma tumors and normal tissues at the mRNA and protein levels. All analyses were measured with the Mann-Whitney U test. **A.** CLIC1 – p = 0.0388, CLIC2 – p = 0.0493, CLIC3 – p < 0.0001, CLIC5 – p < 0.0001. **B.** CLIC3 – p < 0.0001. T – tumor tissue, N – normal tissue. **C.** CLIC1 oral cavity – p = 0.0398, CLIC2 larynx – p = 0.0217, CLIC3 larynx and oral cavity – p < 0.0001, CLIC4 oral cavity – p = 0.0338, CLIC5 oral cavity – p < 0.0001, CLIC6 oral cavity – p = 0.0006. **D.** CLIC3 oral cavity – p < 0.0001.

HNSCC tumors exhibited higher mRNA expression of *CLIC1* (p = 0.0388) and lower expression of *CLIC2*, *CLIC3*, and *CLIC5* (p = 0.0493, p < 0.0001, p < 0.0001, respectively) compared to normal tissue – Fig 1A. CLIC3 protein expression was also significantly lower in HNSCC tumors than in normal tissue (p = 0.0001) – Fig 1B.

Considering tumor location, OSCC tumors showed higher mRNA expression of *CLIC1* and *CLIC4* (p = 0.0398, p = 0.0338, respectively) and lower expression of *CLIC3*, *CLIC5*, and *CLIC6* (p < 0.0001, p < 0.0001, p = 0.0004, respectively) compared to normal tissue – Fig 1C, Table 2. CLIC3 protein expression was decreased in OSCC tumors compared to normal tissue (p < 0.0001) – Fig 1D. In laryngeal squamous cell carcinoma (LSCC) tumors, mRNA expression of *CLIC2* and *CLIC3* was lower than in normal tissue (p = 0.0217, p < 0.0001, respectively) – Fig 1C.

**Table 2:** Chloride intracellular channel family members expression in oral cancer.

	CLIC1	CLIC2	CLIC3	CLIC4	CLIC5	CLIC6
Tumor tissue mRNA	↑	ns	↓	↑	↓	↓
Tumor tissue protein	ns	ni	↓	ns	ni	ni
Blood serum protein	↑	ns	↑	↓	ns	↓

↑ – higher expression in the tumor compared to the normal tissue or in the blood serum of OSCC patients compared to the control group, ↓ – lower expression in the tumor compared to the normal tissue or in the blood serum of OSCC patients compared to the control group, ns – the difference was not significant, ni – the protein expression was not investigated.

Additionally, OSCC tumors had lower *CLIC5* than LSCC tumors (p = 0.0011) – S1 File Supporting Information. Normal oral tissue presented lower expression of *CLIC1* and *CLIC3* (p = 0.0090, p = 0.0430, respectively) and higher expression of *CLIC6* (p = 0.0009) compared to normal laryngeal tissue – S1 File Supporting Information.

### CLICs tissue expression depends on clinical HNSCC features

**Fig 2:**
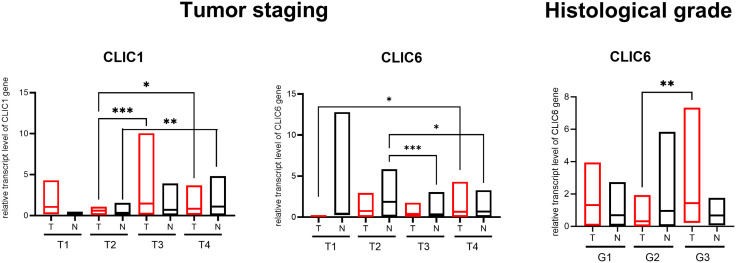
Chloride intracellular channel genes mRNA expression in the head and neck squamous cell carcinoma tumors and normal tissues, depending on the tumor staging and the histological grade. All analyses were measured with the Kruskal-Wallis test and Dunn’s multiple comparison test: Tumor staging-CLIC1 – p = 0.0018, T2 vs. T3 – p = 0.0007, T2 vs. T4 – p = 0.0388; Tumor staging-CLIC6 – p = 0.039, T1 vs. T4 – p = 0.0434; Histological grade-CLIC6 – p = 0.0004, G2 vs. G3 – p = 0.0031.

Tumor (T) staging affected the expression of *CLIC1* and *CLIC6* mRNA (p = 0.0018, p = 0.0390, respectively) – Fig 2. T4 and T3 tumors showed higher *CLIC1* expression than T2 tumors (p = 0.0388, p = 0.0007, respectively), though there was no significant correlation between T staging and *CLIC1* expression (p = 0.1357, r_s_ = 0.1510) – Fig 2. T4 tumors had higher *CLIC6* expression than T1 tumors (p = 0.0434), but no correlation was found between T staging and *CLIC6* expression (p = 0.7459, r_s_ = 0.0366). Histological grade influenced *CLIC6* mRNA expression (p = 0.0004) – Fig 2.

The CLICs expressions varied between the tumor and the normal tissues under different clinical conditions, including age, biological sex, TNM staging, and histological grade. A detailed summary is provided in S1 File Supporting Information.

### Blood serum expression of CLICs differentiates OSCC patients and the control group

**Fig 3:**
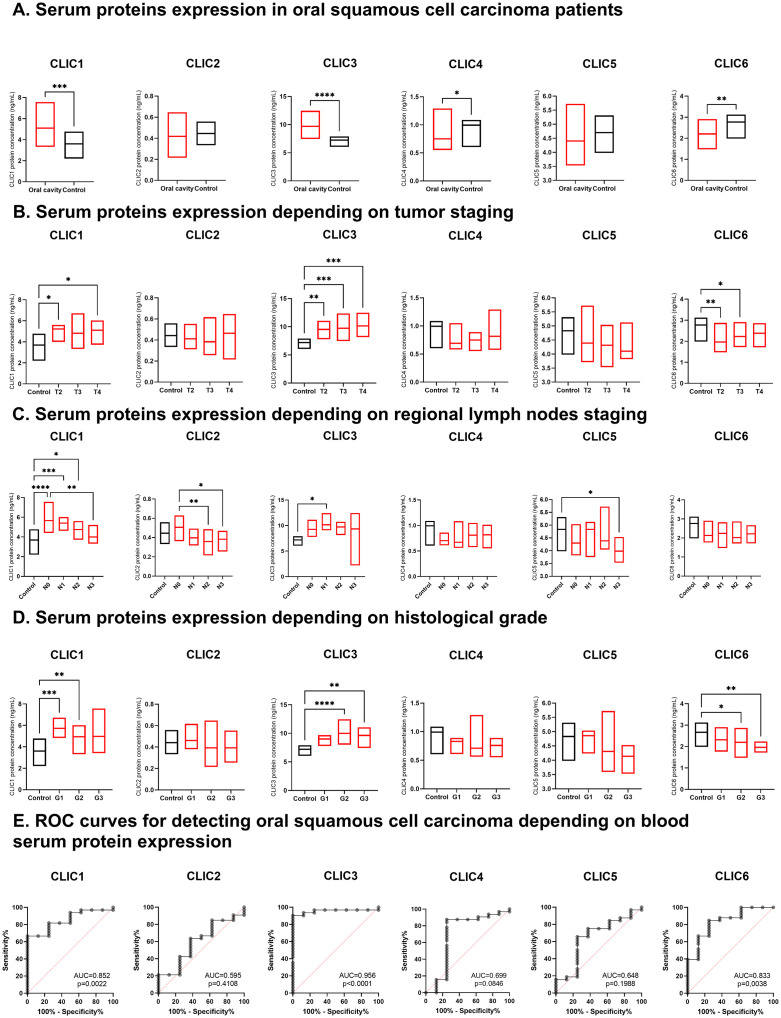
Chloride intracellular channel blood serum protein expression in oral squamous cell carcinoma patients and the control group. **A.** All analyses were measured with the unpaired t-test: CLIC1 – p = 0.0023, CLIC3 – p < 0.0001, CLIC4 – p = 0.0122, CLIC6 – p = 0.0015. **B.** CLIC1 – Kruskal-Wallis test – p = 0.0121, Dunn’s multiple comparison test: Control vs. T2 – p = 0.0228, Control vs. T4 – p = 0.031), CLIC3 – ANOVA – p < 0.0001, Tukey’s multiple comparison test: Control vs. T2 – p = 0.0022, Control vs. T3 – p = 0.0004, Control vs. T4 – p = 0.0001. CLIC6 – ANOVA – p = 0.0055, Tukey’s multiple comparison test: Control vs. T2 – p = 0.0035, Control vs. T3 – p = 0.033. **C.** All analyses were measured with ANOVA and Tukey’s multiple comparisons test: CLIC1 – p = 0 < 0001, Control vs. N0 – p < 0.0001, Control vs. N1 – p = 0.0010, Control vs. N2 – p = 0.0365, N0 vs. N3 p = 0.0049; CLIC2 – p = 0.0082, N0 vs. N2 – p = 0.0082, N0 vs. N3 – p = 0.0377; CLIC3 – p = 0.0281, Control vs. N1 – p = 0.0427; CLIC5 – p = 0.0405, Control vs. N3 – p = 0.0337. **D.** All analyses were measured with ANOVA and Tukey’s multiple comparisons test: CLIC1 – p = 0.0008, Control vs. G1 – p = 0.0006, Control vs. G2 – p = 0.0055; CLIC3 – p < 0.0001, Control vs. G2 – p < 0.0001, Control vs. G3 – p = 0.0034; CLIC6 – p = 0.0046, Control vs. G2 – p = 0.0192, Control vs. G3 – p = 0.0034.

In the blood serum, OSCC patients showed higher expression of CLIC1 and CLIC3 proteins (p = 0.0023, p < 0.0001, respectively) and lower expression of CLIC4 and CLIC6 proteins compared to healthy individuals in the control group (p = 0.0122, p = 0.0015, respectively) – Fig 3A, Table 2.

OSCC patients exhibited increased CLIC1, CLIC3, and decreased CLIC6 protein expression across multiple T stages compared to the control group (p = 0.0121, p < 0.0001, p = 0.0055, respectively); however, no significant differences were observed between T stages – Fig 3B. The N staging influenced the CLIC1 and CLIC2 protein expression (p  < 0.0001, p = 0.0082, respectively), while CLIC3 and CLIC5 protein expression varied between the patients and control group in specific N stages (p = 0.0281, p = 0.0405, respectively) – Fig 3C. OSCC patients also had higher CLIC1, CLIC3, and lower CLIC6 protein expression across different tumor histological grades compared to the control group (p = 0.0008, p < 0.0001, p = 0.0046, respectively), though no differences were found between histological grades – Fig 3D.

The CLICs blood serum expressions varied between OSCC patients and the control group across different clinical conditions, including age, biological sex, TNM staging, and histological grade. A detailed summary is provided in S1 File Supporting Information.

In the ROC curves analysis, AUC (area under the curve) values exceeded 0.8, indicating a strong ability of CLIC1, CLIC3, and CLIC6 proteins to distinguish oral cancer patients and the control group (respectively: AUC = 0.852, p = 0.0022; AUC = 0.956, p < 0.0001; AUC = 0.833, p = 0.0038) – Fig 3E. Based on ROC curve analyses, we determined optimal cut-off levels for CLIC protein serum concentrations to diagnose OSCC with high sensitivity and specificity for CLIC1, CLIC3, and CLIC6 – Table 3.

**Table 3:** The proposed cut-off levels of the chloride intracellular channels protein blood serum concentration, and the corresponding sensitivity and specificity in diagnosing oral squamous cell carcinoma.

	Cut-off (ng/mL)	Se (%)	95% CI (%)	Sp%	95% CI (%)	p-value	AUC
CLIC1	> 4.260	81,82	65,61-91,39	75	40,93-95,56	0,0022	0,8523
CLIC2	< 0.4257	63,64	46,62-77,81	62,5	30,57-86,32	0,4108	0,5947
CLIC3	> 7.959	90,32	75,10-96,65	100	67,56-100,0	<0,0001	0,9556
CLIC4	< 0.9150	87,5	71,93-95,03	75	40,93-95,56	0,0846	0,6992
CLIC5	< 4.580	65,63	48,31-79,59	75	40,93-95,56	0,1988	0,6484
CLIC6	< 2.460	84,85	69,08-93,35	75	40,93-95,56	0,0038	0,8333

Se – sensitivity, Sp – specificity, CI – confidence interval, AUC – area under the curve.

Furthermore, multiple significant correlations between CLIC expression levels were identified in the Spearman correlation matrix (S1 File Supporting Information). *CLIC2* mRNA expression correlated with all other genes, whereas *CLIC3* and *CLIC6* correlated with all except *CLIC1.* In protein analysis, tumor expression of CLIC3 correlated with CLIC4. In blood serum, CLIC2 protein expression correlated with CLIC1, CLIC4, and CLIC6; additionally, CLIC1 correlated with CLIC5, and CLIC4 correlated with CLIC6.

## Discussion

Our study shows that the CLIC family may play a crucial role in HNSCC pathogenesis. We found significant differences in the expression of all CLIC members in HNSCC patients, both in tumors and blood serum, compared to controls. These changes were particularly evident in OSCC, where *CLIC1, CLIC3, CLIC4, CLIC5*, and *CLIC6* showed significant differences at the mRNA level, CLIC3 at the protein level in tumors, and CLIC1, CLIC3, CLIC4, and CLIC6 in the blood serum. Notably, CLIC3 was differently expressed at the mRNA and protein levels in all studied OSCC tissues, highlighting its potential as promising OSCC biomarker. In LSCC tumors, we found lower *CLIC2* and *CLIC3* mRNA expression compared to normal tissue. Our findings are novel, as limited studies have examined CLICs in HNC [31–33,38–46] Table 4.

**Table 4:** Original studies regarding chloride intracellular channels in head and neck cancer.

Chloride intracellular channel	Author	Year	Cancer and study type	Studied population	Conclusion
CLIC1-CLIC6	Current study Wojtera et al.	2025	OSCC LSCC Clinical	Tumors – n = 99 Blood – n = 38	CLIC1, CLIC3, CLIC4, and CLIC6 are promising candidates for HNSCC/OSCC biomarkers and/or therapeutic targets
CLIC1					
	Wojtera et al. [33]	2022	OSCC LSCC Clinical	Blood – n = 20	CLIC1 plasmatic expression may be associated with regional metastases in OSCC
	Feng et al. [31]	2019	OSCC *In vitro*	SCC-15 cell culture	CLIC1 may promote the progression of OSCC by activating MAPK/ERK and MAPK/p38 pathways
	Xu et al. [38]	2018	OSCC Clinical	Tissue – n = 72 Blood – n = 54	CLIC1 may be a diagnostic biomarker and/or therapeutic target in OSCC management
	Wang et al. [37]	2018	NPC *In vitro*	CNE1, CNE2 cell cultures	CLIC1 may play a role in the mechanisms of chemoresistance in NPC
	Cristofaro et al. [40]	2014	OSCC (gingiva) Clinical	Tumor – n = 3	CLIC1 may be a diagnostic biomarker and/or therapeutic target in OSCC management
	Karsani et al. [43]	2014	OSCC *In vitro*	Cell cultures established from cancerous tissues	CLIC1 may play a role in OSCC development and progression
	Kim et al. [36]	2010	LSCC *In vitro*	HEp-2/RR-Hep2 cell cultures	CLIC1 may play a role in the mechanisms of radioresistance in LSCC
	Chang et al. [39]	2009	NPC Clinical *In vitro*	Tumor – n = 40 Blood – n = 74 NPC-TW04 cell culture	CLIC1 may be a diagnostic biomarker and/or therapeutic target in NPC management
CLIC3					
	Wang et al. [44]	2015	MEC Clinical	Tumor – n = 105	*CLIC3* and its methylation may be an epigenetic biomarker and/or therapeutic target in salivary MEC
CLIC4					
	Xerez et al. [45]	2023	OSCC OSV Clinical	Tumor – n = 35	CLIC4 may impact the biological behavior difference between OSCC and OSV
	Lima et al. [32]	2020	LLSCC Clinical	Tumor – n = 50	CLIC4 may be a diagnostic biomarker and/or therapeutic target in LLSCC management
	Zhu et al. [35]	2020	NPC Clinical *In vitro*	Tumor – n = 6 CNE1, CNE2 cell cultures	CLIC4 may play a role in the mechanism of radioresistance in NPC and may be the NPC predictive biomarker for NPC prognosis and recurrence
	Carofino et al. [42]	2019	OSCC *In vitro*	SCC4, SCC9, SCC15, SCC25 cell cultures	*CLIC4* is regulated by miR-142-3p; CLIC4 expression in tumors is lower in epithelial cells than in stromal fibroblasts and endothelial cells
	Xue et al. [41]	2016	OSCC Clinical *In vitro*	Tumor – n = unknown HN4 cell culture	CLIC4 may be a diagnostic biomarker and/or therapeutic target in OSCC management
CLIC6					
	Liu et al. [46]	2024	NPC Clinical	Tumor – n = 40	*CLIC6* may be a diagnostic biomarker and/or therapeutic target in NPC management

LLSCC – lower lip squamous cell carcinoma, LSCC – laryngeal squamous cell carcinoma, MEC – mucoepidermoid salivary gland carcinoma, NPC – nasopharyngeal carcinoma, OSCC – oral squamous cell carcinoma, OSV – oral verrucous carcinoma.

### CLIC1

CLIC1 has been extensively studied in various malignancies, with particular emphasis on tumors of the nervous, respiratory, digestive, reproductive, and urinary systems [18]. It plays a significant role in tumor formation and progression: in pancreatic cancer, CLIC1 functions as an oncogene; in gallbladder and gastric cancers, it promotes cell proliferation through MAPK/AKT signaling and facilitates the formation of tumor-associated fibroblasts; in colorectal cancer, it modulates cell volume and reactive oxygen species (ROS) levels; and in glioblastoma stem cells, it promote cellular proliferation and G1/S cell cycle transition [30]. CLIC1 has been proposed as a tumor biomarker in a wide range of cancers, including bladder, breast, cervical, colorectal, esophageal, gallbladder, gastric, hepatic, pulmonary, nasopharyngeal, oral, ovarian, pancreatic, and renal cancers, as well as gliomas [17,19,20]. Notably, its detectable expression in peripheral blood suggests its potential utility as a non-invasive biomarker for chronic lymphocytic leukemia, NPC, OSCC, and ovarian cancer [17,33,38,39,48]. Despite substantial evidence supporting the diagnostic and prognostic relevance of *CLIC1* across multiple cancer types, it has yet to be integrated into routine clinical oncology practice.

Despite numerous studies on *CLIC1* in oncology, HNSCC received marginal attention [17]. Chang et al. initiated research in this area in 2009 by identifying CLIC1 as a potential biomarker, finding elevated CLIC1 protein expression in the blood plasma of NPC patients and variations based on TNM staging [39]. Afterward, Karsani et al. suggested CLIC1 may be involved in OSCC development and progression [43]. Independently, Cristofaro et al. identified 17 overexpressed proteins in gingival squamous cell carcinoma tissues, with CLIC1 being the most promising [40]. Xu et al. confirmed increased CLIC1 protein expression in OSCC tissues and correlated it with TNM staging, histological grade, and OS. They also found higher CLIC1 expression in the blood plasma of OSCC patients [38]. Finally, our previous study suggested that CLIC1 plasmatic expression may be associated with regional metastases in OSCC [33].

An *in vitro* study by Feng et al. confirmed that CLIC1 may promote OSCC progression by specific activation of MAPK/ERK and MAPK/p38 pathways [31]. Additionally, CLIC1 may influence radio- and chemoresistance in HNSCC. Kim et al. found that CLIC1 suppression leads to radioresistance of LSCC by inhibiting the ROS production [36], while Wang et al. suggested that CLIC1 contributes to chemoresistance mechanisms in NPC [37].

In our study, *CLIC1* mRNA was overexpressed in the OSCC tumors, corresponding with increased CLIC1 levels in the blood serum of OSCC patients, consistent with previous OSCC research [38,40]. However, we found no significant difference in tumor protein expression, which may be due to the limited study population and low protein concentration isolated from tissue samples. More advanced tumors showed higher *CLIC1* mRNA expression. Interestingly, normal oral tissue had lower expression of *CLIC1* mRNA than the laryngeal – S1 File Supporting Information.

### CLIC2

Research on CLIC2 in oncology remains limited, and to date, no studies have specifically investigated its role in HNSCC. CLIC2 has been shown to inhibit matrix metalloproteinase 14 (MMP14) activity, thereby suppressing invasion and metastasis in gliomas [49]. Ozaki et al. proposed that CLIC2 may play a preventive role in tumor invasion and metastasis and could serve as a therapeutic target [13]. In contrast, Xu et al. found that *CLIC2* is co-expressed with PD-1 and PD-L1, correlating with tumor-infiltrating lymphocytes and the prognosis of breast cancer patients [50]. Additionally, Yingjuan et al. identified *CLIC2* as a part of a ten-gene prognostic signature developed for skin melanoma [51].

In our study, *CLIC2* mRNA level was down-expressed in LSCC tumors compared to normal tissue. Although there was no significant difference in CLIC2 blood protein expression between OSCC patients and the control group, its expression varied with N staging – Fig 3C. This is the first study to bring insights into CLIC2 in HNSCC.

### CLIC3

CLIC3 promotes the progression of bladder cancer, interacting with NAT10 and modifying p21 mRNA [52]*.* Furthermore, *CLIC3* has been identified as a prognostic biomarker in several malignancies, including cancers of the esophagus [53], stomach [54], pancreas [19], lung [55], and bladder [22,56]. Its high expression has also been linked to advanced stages of HCC [20]. While CLIC3 appears to play a notable role across multiple cancer types, current evidence is limited to individual studies and lacks clinical validation.

Regarding HNC, CLIC3 has previously been investigated in mucoepidermoid salivary gland carcinoma (MEC). Wang et al. found that MEC tumors exhibited hypomethylation of the *CLIC3* promoter and increased CLIC3 protein expression compared to normal salivary gland tissue [44]. However, this is the first study that explores the role of CLIC3 in HNSCC. In our findings, *CLIC3* mRNA was down-expressed in OSCC and LSCC tumors compared to normal tissue, while normal oral mucosa had lower *CLIC3* mRNA expression than normal laryngeal mucosa. CLIC3 protein expression was higher in the blood serum of OSCC patients compared to the control group. The lower expression of CLIC3 in tumor tissues and higher expression in the blood suggest that OSCC cells may secrete CLIC3 into the bloodstream. Strong statistical associations suggesting that CLIC3 may be the most promising biomarker and/or therapeutic target highlight the need for further studies of CLIC3 in HNSCC, especially in the liquid biopsy approach. Nevertheless, these findings may be biased by the limited size of the study (n = 38) and control group (n = 8).

### CLIC4

CLIC4 is involved in the pathogenesis of multiple cancers. In breast and ovarian cancers, it promotes tumor progression via TGF-β–mediated ROS production. In colorectal cancer, CLIC4 signaling overlaps with nine key pathways: nuclear receptor (NR), VEGF, NRF2, MAPK, PI3K/AKT, insulin, IL-18, and microRNA regulatory pathways. In gastric cancer, its overexpression inhibits CD44 and OCT4, reducing migration, invasion, and epithelial-mesenchymal transition (EMT). In lung cancer, CLIC4 promotes metastasis by impairing endothelial integrity through the ERM/PAR1 pathway. In gliomas, it may inhibit apoptosis triggered by oxidative, mitochondrial, and endoplasmic reticulum stress [24].

Furthermore, CLIC4 has shown potential as a diagnostic and prognostic indicator in ovarian, pancreatic, and esophageal malignancies [24]. In ovarian cancer, it may complement CA125 in serological detection [48,57], while in pancreatic and esophageal cancer, it has been associated with disease prognosis [19,53]. Although these findings are promising, they are primarily derived from *in silico* analyses, *in vitro* assays, and animal studies, with limited validation in clinical settings.

*CLIC4* is the second most studied member of the CLIC family in relation to HNSCC. Xue et al. found higher *CLIC4* expression in OSCC tissues compared to the normal gingival tissues, and *CLIC4* knockdown led to ATP-induced apoptosis of HN4 cells via ER and mitochondrial pathways [41]. Lima et al. reported that a shift in CLIC4 expression from nuclear to cytoplasmatic localization was associated with lower lip squamous cell carcinoma (LLSCC) progression and changes in p53, TGF-β, and TNF-α expression [32]. Unlike *CLIC1*, higher *CLIC4* expression increases HNSCC radioresistance by affecting intracellular nitric oxide (NO) in the NPC *in vitro* model. Zhu et al. suggested that *CLIC4* may serve as a predictive biomarker for NPC prognosis and local recurrence [35]. Additionally, *CLIC4* expression in tumors is lower in epithelial cells than in stromal fibroblasts and endothelial cells, which is critical when studying bulky tumors [42]. Finally, Xerez et al. found higher CLIC4 expression in OSCC tissues than in oral verrucous carcinoma (OVC), speculating that this may contribute to differences in biological behavior between histological types of oral cancer [45].

In our study, *CLIC4* mRNA was overexpressed in OSCC tumors compared to normal tissue, consistent with the findings of Xue et al. [41]. CLIC4 protein expression was higher in tumors than in normal tissue, but only in men. In the blood serum, CLIC4 protein expression was lower in OSCC patients compared to the control group.

### CLIC5

CLIC5 has been proposed as a biomarker for malignancies of the liver, ovary, lung, and pancreas [25,26,58,59]. In ovarian cancer, it plays a key role in shaping the TME; specifically, *CLIC5* overexpression is negatively associated with CD8 ⁺ T-cell infiltration and positively associated with M2 macrophages (CD163). It has also been linked to tumor development, progression, and malignant potential in ovarian cancer [26]. CLIC5 is notably overexpressed and hypomethylated in pancreatic cancer tissues within the Indian population [58]. In HCC, it contributes to cellular migration and invasion and has been proposed as a prognostic indicator [25]. In lung adenocarcinoma, *CLIC5* expression reflects patients’ prognosis and immune infiltration [59]. However, these observations are based on individual studies and lack broader validation.

According to Huang et al. *CLIC5* may play a crucial role in pan-cancer development, including HNSCC [26]. However, our study is the first to provide insights into the role of *CLIC5* in HNSCC. *CLIC5* mRNA expression was lower in OSCC tumors than in normal tissues and LSCC tumors. In blood serum, CLIC5 protein expression significantly differed between the control group and patients with regional lymph node metastases at the N3 stage.

### CLIC6

Multiple bioinformatic studies have highlighted *CLIC6* as a gene of prognostic interest, often in conjunction with other markers, in prostate cancer [60], breast cancer [29], and lung adenocarcinoma [61]. In HCC, *CLIC6*-based therapy promotes apoptosis, leading to cleaved caspase-3 overexpression and increasing the Bax/Bcl-2 ratio. It also modulates the TME by altering the levels of various cytokines, including IL-1β, IL-4, IL-6, IL-17A, TNF-α, TGF-β, and interferon-γ. Additionally, it helps restore antioxidant enzyme levels while reducing serum markers of oxidative stress, such as MDA and NO [28]. Nevertheless, current evidence remains preliminary and is largely derived from computational analyses, underscoring the need for clinical validation.

CLIC6 has received the least attention in clinical oncology research [17]. A recent study by Liu et al. identified *CLIC6, TPPP3*, and *MUC4* as genes involved in NPC pathogenesis [46]. In our study, *CLIC6* mRNA expression was lower in OSCC tumors than in normal tissues and was associated with T stage and histological grade. OSCC patients also had lower CLIC6 blood serum protein expression than the control group.

### CLICs as liquid biopsy targets for OSCC

Multiple significant differences in the blood serum expression of CLIC proteins in OSCC suggest they may serve as viable liquid biopsy targets. According to Alix-Panabières et al. liquid biopsy enables early diagnosis, tumor staging, prediction of metastatic progression, and treatment monitoring [62]. Our findings provide preliminary evidence supporting the first three of these aspects in relation to CLICs in HNSCC. We observed higher expression of CLIC1 and CLIC3 proteins and lower expression of CLIC4 and CLIC6 proteins in the blood serum of OSCC patients compared to the control group. Additionally, regional lymph node metastases influenced the blood serum expression of CLIC1 and CLIC2 proteins – Fig 3C. However, recent review articles have overlooked CLICs as potential HNSCC biomarkers [63–65], highlighting the need for further research to establish their clinical relevance. While recent studies have focused on innovative biomarkers, such as circulating tumor cells (CTC), circulating tumor DNA (ctDNA), extracellular vesicles, and circulating immune cells [65], proteomics remains a crucial area for identifying liquid biopsy targets [66].

### CLIC3 is a promising candidate for the OSCC biomarker

As the expression patterns suggest the involvement of CLICs in OSCC pathogenesis, we summarized their expression at different levels in Table 3. This is the first study to analyze the entire CLIC family and identify novel OSCC biomarkers. Given the promising AUC results for CLIC3 in detecting OSCC, along with its significantly higher protein expression in the blood serum and significantly lower mRNA and protein expression in tumor tissues, we speculate that CLIC3 is the most promising OSCC biomarker – Figs 1A, 1B, 1C, 1D and 3A, 3E, Table 3.

### The future of CLICs in HNSCC

This study provides a solid foundation for further research on CLIC expression in a larger population. Multicenter, high-volume studies could facilitate the validation of CLICs as HNSCC biomarkers. Future research should focus on the CLICs expression following the treatment and in the event of cancer recurrence to assess their potential value for oncological monitoring. Additionally, more *in vitro* studies are needed to develop targeted therapeutic strategies.

### Study limitations

We acknowledge certain limitations in our study. We omitted the analysis of CLIC2, CLIC5, and CLIC6 proteins in tumor tissues due to challenges with reliable antibody optimization. The Western Blot technique was used for tumor tissue CLICs protein analysis to align with available funding. The study population for blood expression analysis was limited (patients – n = 38, control group – n = 8) due to funding constraints. Also, for future perspectives, it is worth including the IHC staining of selected CLICs to correlate the importance of its cellular localization (with particular emphasis on nucleus and cytoplasm) with clinical features and treatment response.

## Conclusions

This is the first study to assess the expression of the entire CLIC family in HNSCC and correlate it with the clinical characteristics, revealing significant associations. Considering the distinct expression patterns of CLIC family members in OSCC, we conclude that CLIC1, CLIC3, CLIC4, and CLIC6 emerge as key players warranting further investigation. They hold potential as OSCC biomarkers, including liquid biopsy, and as therapeutic targets in personalized oncology.

## Supporting information

S1 File Supporting information. (PDF)

S2 File The original blots of examples presented in Fig 1. (PDF)

S3 File Experimental data. (XLSX)
