# Supplementary material for: The pursuit of novel head and neck cancer biomarkers – tissue and blood expression of chloride intracellular channels family
Source: PLoS One. 2025 Oct 24;20(10):e0333487. doi: 10.1371/journal.pone.0333487 (PMC12551828; doi:10.1371/journal.pone.0333487)

Supportive Information Table 1. Primers used in the analysis of the *CLIC* family and *GAPDH* mRNA expression.

Gene	Forward primer	Reverse primer
CLIC1	CAGACCACAGTCCCAGCAAC	CCATCACTGCCAGCCTTCAC
CLIC2	GTACCAATCCTCCGTTCCTGG	CTCAGGTGAGGGTACCTTGGA
CLIC3	CTGCACATCGTCGACACGG	ACACCCTCACTCCCGACAAA
CLIC4	GTTGACCTGAAAAGGAAGCCA	CTTCATTAGCCTCTGGCCTTG
CLIC5	TTCATCAACCCAGGACCAGC	TCCATCGATTCCAGCCTTCAC
CLIC6	TCACCCTCTTCGTCAAGGTAA	AGAGACGCTGAGAAAACGGG
GAPDH	GTCTCCTCTGACTTCAACAGCG	ACCACCCTGTTGCTGTAGCCAA

Supportive Information Table 2. Comparison of chloride intracellular channel mRNA expression between head and neck squamous cell carcinoma tumors and tissues harvested from the free surgical margin, depending on demographic, clinical, and pathological features.

	CLIC1		*CLIC2*		*CLIC3*		*CLIC4*		*CLIC5*		*CLIC6*	
	Mean/Median	*p*-Value	Mean/Median	*p*-Value	Mean/Median	*p*-Value	Mean/Median	*p*-Value	Mean/Median	*p*-Value	Mean/Median	*p*-Value
Oral cavity	tumor=0.71<br>normal=0	0.0398	tumor=0.43<br>normal=0.67	ns	tumor=0.15<br>normal=1.04	<0.0001	tumor=0.65<br>normal=0.33	0.0338	tumor=0.26<br>normal=2.16	<0.0001	tumor=0.49<br>normal=1.30	0.0004
Larynx	tumor=0.87<br>normal=0.96	ns	tumor=0.45<br>normal=1.01	0.0217	tumor=0.22<br>normal=1.69	<0.0001	tumor=0.67<br>normal=0.52	ns	tumor=1.06<br>normal=0.77	ns	tumor=0.65<br>normal=0.50	ns
Age ≤ 60	tumor=0.80<br>normal=0.66	ns	tumor=0.96<br>normal=0.74	ns	tumor=0.16<br>normal=1.09	<0.0001	tumor=1.15<br>normal=0.99	ns	tumor=0.53<br>normal=2.19	0.0015	tumor=1.86<br>normal=1.02	0.0437
Age > 60	tumor=0.83<br>normal=0.67	ns	tumor=0.42<br>normal=0.80	0.0058	tumor=0.20<br>normal=1.68	<0.0001	tumor=0.53<br>normal=0.32	ns	tumor=0.38<br>normal=0.84	0.0006	tumor=0.30<br>normal=0.50	ns
Female	tumor=0.78<br>normal=0.95	ns	tumor=0.63<br>normal=1.28	ns	tumor=0.14<br>normal=2.42	<0.0001	tumor=0.86<br>normal=0.69	ns	tumor=0.30<br>normal=3.18	<0.0001	tumor=0.68<br>normal=3.90	0.0002
Male	tumor=0.82<br>normal=0.62	0.0487	tumor=0.44<br>normal=0.47	ns	tumor=0.24<br>normal=0.94	<0.0001	tumor=0.56<br>normal=0.34	ns	tumor=0.42<br>normal=0.84	0.0025	tumor=0.52<br>normal=0.50	ns
Stage I & Stage II	tumor=0.69<br>normal=0.32	ns	tumor=0.53<br>normal=0.40	ns	tumor=0.08<br>normal=0.78	0.0012	tumor=0.65<br>normal=0.34	ns	tumor=0.30<br>normal=2.27	0.0041	tumor=0.68<br>normal=1.27	ns
Stage III	tumor=1.09<br>normal=0.73	ns	tumor=0.24<br>normal=0.80	ns	tumor=0.24<br>normal=1.04	0.0154	tumor=0.55<br>normal=0.23	ns	tumor=0.19<br>normal=0.62	0.0242	tumor=0.26<br>normal=0.34	ns
Stage IV	tumor=0.80<br>normal=0.95	ns	tumor=0.63<br>normal=1.18	ns	tumor=0.21<br>normal=2.31	<0.0001	tumor=0.58<br>normal=0.78	ns	tumor=0.48<br>normal=0.94	0.0025	tumor=0.65<br>normal=0.88	ns
T1&T2	tumor=057<br>normal=0.33	ns	tumor=0.33<br>normal=0.34	ns	tumor=0.12<br>normal=0.94	<0.0001	tumor=0.52<br>normal=0.39	ns	tumor=0.28<br>normal=2.27	<0.0001	tumor=0.52<br>normal=1.66	0.0099
T3	tumor=1.45<br>normal=0.70	0.0272	tumor=0.66<br>normal=0.66	ns	tumor=0.34<br>normal=1.27	0.0404	tumor=0.72<br>normal=0.17	ns	tumor=0.48<br>normal=0.47	ns	tumor=0.37<br>normal=0.34	ns
T4	tumor=0.83<br>normal=1.09	ns	tumor=0.55<br>normal=1.28	0.0154	tumor=0.12<br>normal=2.14	<0.0001	tumor=0.56<br>normal=0.86	ns	tumor=0.57<br>normal=0.89	0.0370	tumor=0.65<br>normal=0.68	ns
N0	tumor=0.74<br>normal=0.59	ns	tumor=0.28<br>normal=0.64	ns	tumor=0.22<br>normal=0.86	0.0010	tumor=0.63<br>normal=0.54	ns	tumor=0.38<br>normal=1.13	0.0024	tumor=0.52<br>normal=0.68	ns
N1	tumor=0.89<br>normal=0.79	ns	tumor=0.25<br>normal=1.22	0.0407	tumor=0.11<br>normal=0.99	0.0001	tumor=0.79<br>normal=0.48	ns	tumor=0.45<br>normal=1.33	0.0186	tumor=0.22<br>normal=0.48	ns
N2	tumor=0.80<br>normal=0.81	ns	tumor=0.96<br>normal=1.6	ns	tumor=0.45<br>normal=2.62	0.0002	tumor=0.58<br>normal=0.66	ns	tumor=0.41<br>normal=1.10	ns	tumor=0.73<br>normal=1.01	ns
N3	tumor=1.54<br>normal=0.37	ns	tumor=0.59<br>normal=0.23	ns	tumor=0.27<br>normal=0.83	ns	tumor=0.27<br>normal=0.17	ns	tumor=0.90<br>normal=0.29	ns	tumor=1.75<br>normal=0.34	ns
G1	tumor=0.99<br>normal=0.68	ns	tumor=0.30<br>normal=0.61	ns	tumor=0.74<br>normal=1.12	ns	tumor=0.45<br>normal=0.31	ns	tumor=0.26<br>normal=0.59	ns	tumor=1.31<br>normal=0.68	ns
G2	tumor=0.74<br>normal=0.70	ns	tumor=0.62<br>normal=0.73	ns	tumor=0.11<br>normal=1.68	<0.0001	tumor=0.63<br>normal=0.55	ns	tumor=0.48<br>normal=1.59	<0.0001	tumor=0.32<br>normal=1.00	0.0010
G3	tumor=1.48<br>normal=0.50	ns	tumor=0.54<br>normal=0.46	ns	tumor=0.35<br>normal=1.70	ns	tumor=0.79<br>normal=0.36	ns	tumor=0.38<br>normal=1.09	0.0413	tumor=1.44<br>normal=0.68	ns

Depending on the normality test by Shapiro-Wilk, the unpaired t-test or the Mann-Whitney U-test was used in analyses.

Supportive Information Table 3. Comparison of chloride intracellular channel protein expression between head and neck squamous cell carcinoma tumors and tissues harvested from the free surgical margin, depending on demographic, clinical, and pathological features.

	CLIC1 Mean/Median	CLIC1 *p*-Value	CLIC3 Mean/Median	CLIC3 *p*-Value	CLIC4 Mean/Median	CLIC4 *p*-Value
Oral cavity	tumor=0.30<br>normal=0.35	ns	tumor=0.23<br>normal=1.00	<0.0001	tumor=0.27<br>normal=0.42	ns
Larynx	tumor=0.06<br>normal=0.13	ns	tumor=0.10<br>normal=0.86	ns	tumor=0.10<br>normal=0.13	ns
Age ≤ 60	tumor=0.17<br>normal=0.13	ns	tumor=0.27<br>normal=1.20	0.0011	tumor=0.33<br>normal=0.32	ns
Age > 60	tumor=0.27<br>normal=0.40	ns	tumor=0.24<br>normal=0.86	0.0204	tumor=0.17<br>normal=0.38	ns
Female	tumor=0.37<br>normal=0.91	ns	tumor=0.42<br>normal=0.57	ns	tumor=0.45<br>normal=0.16	ns
Male	tumor=0.17<br>normal=0.15	ns	tumor=0.18<br>normal=0.96	0.0001	tumor=0.24<br>normal=0.38	0.02
Stage I & Stage II	tumor=0.43<br>normal=1.46	ns	tumor=0.17<br>normal=1.91	0.0205	tumor=0.48<br>normal=1.69	ns
Stage III	tumor=0.40<br>normal=0.38	ns	tumor=0.37<br>normal=0.46	ns	tumor=0.06<br>normal=0.35	0.0085
Stage IV	tumor=0.25<br>normal=0.21	ns	tumor=0.23<br>normal=0.94	0.0013	tumor=0.33<br>normal=0.16	ns
T1 & T2	tumor=0.42<br>normal=0.68	ns	tumor=0.25<br>normal=1.06	0.0172	tumor=0.35<br>normal=1.69	0.0222
T3	tumor=0.37<br>normal=0.55	ns	tumor=0.23<br>normal=0.96	0.0106	tumor=0.24<br>normal=0.15	ns
T4	tumor=0.11<br>normal=0.13	ns	tumor=0.45<br>normal=0.87	ns	tumor=0.28<br>normal=0.16	ns
N0	tumor=0.19<br>normal=0.38	ns	tumor=0.26<br>normal=1.01	ns	tumor=0.13<br>normal=0.24	ns
N1	tumor=0.49<br>normal=0.29	ns	tumor=0.45<br>normal=1.01	ns	tumor=0.16<br>normal=0.45	ns
N2	tumor=0.22<br>normal=0.13	ns	tumor=0.39<br>normal=0.91	0.0469	tumor=0.40<br>normal=0.27	ns
N3	tumor=0.55<br>normal=0.99	ns	tumor=0.12<br>normal=0.57	ns	tumor=0.39<br>normal=2.80	ns
G1	tumor=0.25<br>normal=0.23	ns	tumor=0.72<br>normal=0.83	ns	tumor=0.21<br>normal=0.53	ns
G2	tumor=0.36<br>normal=0.21	ns	tumor=0.24<br>normal=0.89	0.0183	tumor=0.24<br>normal=0.35	ns
G3	tumor=0.23<br>normal=0.44	ns	tumor=0.23<br>normal=1.88	0.0025	tumor=0.42<br>normal=0.23	ns

Depending on the normality test by Shapiro-Wilk, the unpaired t-test or the Mann-Whitney U-test was used in analyses.

Supportive Information Table 4. Comparison of chloride intracellular channel blood serum protein expression between oral squamous cell carcinoma patients and the control group, depending on demographic, clinical, and pathological features.

	CLIC1		CLIC2		CLIC3		CLIC4		CLIC5		CLIC6	
	Mean/Median	*p*-Value	Mean/Median	*p*-Value	Mean/Median	*p*-Value	Mean/Median	*p*-Value	Mean/Median	*p*-Value	Mean/Median	*p*-Value
Oral cancer vs. Control group	patients=5.09<br>control=3.61	0.0002	patients=0.42<br>control=0.44	ns	patients=9.78<br>control=7.19	<0.0001	patients=0.75<br>control=0.99	0.0122	patients=4.40<br>control=4.70	ns	patients=2.16<br>control=2.66	0.0015
Age ≤ 60	patients=4.82<br>control=3.71	0.0200	patients=0.38<br>control=0.43	ns	patients=9.24<br>control=7.26	0.0006	patients=0.68<br>control=0.99	ns	patients=4.42<br>control=4.83	ns	patients=2.12<br>control=2.77	0.0050
Age > 60	patients=5.36<br>control=3.42	0.0055	patients=0.40<br>control=0.53	ns	patients=9.23<br>control=7.19	0.0029	patients=0.81<br>control=0.97	ns	patients=4.39<br>control=4.48	ns	patients=2.24<br>control=2.49	ns
Female	patients=5.15<br>control=3.36	ns	patients=0.38<br>control=0.38	ns	patients=9.24<br>control=7.18	ns	patients=0.67<br>control=0.60	ns	patients=4.38<br>control=4.47	ns	patients=1.98<br>control=2.43	ns
Male	patients=5.21<br>control=3.70	0.0029	patients=0.43<br>control=0.46	ns	patients=10.02<br>control=7.20	<0.0001	patients=0.78<br>control=1.00	0.0025	patients=4.41<br>control=4.84	ns	patients=2.21<br>control=2.94	0.0052
Stage II	patients=6.02<br>control=3.61	0.0080	patients=0.46<br>control=0.44	ns	patients=9.15<br>control=7.19	0.0036	patients=0.67<br>control=0.99	ns	patients=4.31<br>control=4.70	ns	patients=1.98<br>control=2.66	0.0082
Stage III	patients=5.46<br>control=3.61	0.0004	patients=0.44<br>control=0.44	ns	patients=9.48<br>control=7.19	<0.0001	patients=0.71<br>control=0.99	0.0221	patients=4.61<br>control=4.70	ns	patients=2.16<br>control=2.66	0.0464
Stage IV	patients=4.79<br>control=3.61	0.0025	patients=0.40<br>control=0.44	ns	patients=10.00<br>control=7.19	<0.0001	patients=0.81<br>control=0.99	0.0356	patients=4.33<br>control=4.70	ns	patients=2.22<br>control=2.66	0.0065
T2	patients=5.22<br>control=3.69	0.0019	patients=0.41<br>control=0.44	ns	patients=9.52<br>control=7.19	<0.0001	patients=0.69<br>control=0.99	0.0418	patients=4.51<br>control=4.70	ns	patients=2.02<br>control=2.66	0.0025
T3	patients=4.74<br>control=3.69	ns<br>(p=0.0502)	patients=0.40<br>control=0.44	ns	patients=9.73<br>control=7.19	<0.0001	patients=0.75<br>control=0.99	0.0056	patients=4.42<br>control=4.70	ns	patients=2.19<br>control=2.66	0.0060
T4	patients=5.04<br>control=3.61	0.0021	patients=0.45<br>control=0.44	ns	patients=10.12<br>control=7.19	0.0003	patients=0.85<br>control=0.99	ns	patients=4.28<br>control=4.70	ns	patients=2.32<br>control=2.66	ns
N0	patients=5.86<br>control=3.61	0.0004	patients=0.51<br>control=0.44	ns	patients=9.25<br>control=7.19	0.0003	patients=0.70<br>control=0.99	0.0205	patients=4.14<br>control=4.70	ns	patients=2.13<br>control=2.66	0.0143
N1	patients=5.29<br>control=3.61	0.0003	patients=0.40<br>control=0.44	ns	patients=10.14<br>control=7.19	0.0001	patients=0.67<br>control=0.99	ns	patients=4.61<br>control=4.70	ns	patients=2.20<br>control=2.66	0.0435
N2	patients=4.80<br>control=3.61	0.0098	patients=0.36<br>control=0.44	ns	patients=9.71<br>control=7.19	<0.0001	patients=0.81<br>control=0.99	0.0211	patients=4.56<br>control=4.70	ns	patients=2.20<br>control=2.66	0.0363
N3	patients=3.75<br>control=3.61	ns	patients=0.38<br>control=0.44	ns	patients=9.34<br>control=7.19	ns	patients=0.81<br>control=0.99	0.0401	patients=3.96<br>control=4.70	ns	patients=2.17<br>control=2.66	0.0121
G1	patients=5.73<br>control=3.61	0.0007	patients=0.47<br>control=0.44	ns	patients=9.01<br>control=7.19	0.0010	patients=0.83<br>control=0.99	0.0480	patients=4.78<br>control=4.70	ns	patients=2.32<br>control=2.66	ns
G2	patients=4.94<br>control=3.61	0.0003	patients=0.41<br>control=0.44	ns	patients=9.62<br>control=7.19	0.0045	patients=0.71<br>control=0.99	0.0408	patients=4.38<br>control=4.70	ns	patients=2.20<br>control=2.66	0.0073
G3	patients=4.96<br>control=3.61	ns	patients=0.44<br>control=0.44	ns	patients=9.63<br>control=7.19	0.0005	patients=0.76<br>control=0.99	ns	patients=4.04<br>control=4.70	0.0269	patients=1.98<br>control=2.66	0.0019

Depending on the normality test by Shapiro-Wilk, the unpaired t-test or the Mann-Whitney U-test was used in analyses.

Supportive Information Table 5. Spearman correlation matrix of *CLICs* mRNA expression in HNSCC tumors – *p* values.

	CLIC1	CLIC2	CLIC3	CLIC4	CLIC5	CLIC6
CLIC1		0,003492724	0,086379423	0,409295	0,545019	0,938014
CLIC2	0,003492724		5,12429E-06	0,000439	1,17E-07	1,76E-05
CLIC3	0,086379423	5,12429E-06		0,000152	0,163453	7,29E-05
CLIC4	0,409295021	0,000438683	0,00015184		0,010327	0,001073
CLIC5	0,545019483	1,16535E-07	0,163452797	0,010327		0,000599
CLIC6	0,938013513	1,75783E-05	7,28885E-05	0,001073	0,000599	

Supportive Information Table 6. Spearman correlation matrix of CLIC*s* protein expression in HNSCC tumors – *p* values.

	CLIC1	CLIC3	CLIC4
CLIC1		0,057786798	0,117276043
CLIC3	0,057786798		0,000118938
CLIC4	0,117276043	0,000118938	

Supportive Information Table 7. Spearman correlation matrix of CLIC*s* protein expression in blood serum of HNSCC patients – *p* values.

	CLIC1	CLIC2	CLIC3	CLIC4	CLIC5	CLIC6
CLIC1		5,31317E-06	0,224309734	0,227073	0,004147	0,053146
CLIC2	5,31E-06		0,195310042	9,16E-06	0,196591	0,000138
CLIC3	0,224309734	0,195310042		0,086115	0,22795	0,221938
CLIC4	0,227073457	9,1633E-06	0,086115289		0,417973	5,06E-05
CLIC5	0,004146844	0,196590649	0,227949581	0,417973		0,400712
CLIC6	0,053146373	0,000137774	0,221937572	5,06E-05	0,400712	

Supportive Information Figure 1. Expression of *CLIC1-CLIC6* mRNA – a comparison between oral and laryngeal squamous cell carcinoma tumors and between normal oral and laryngeal mucosa.

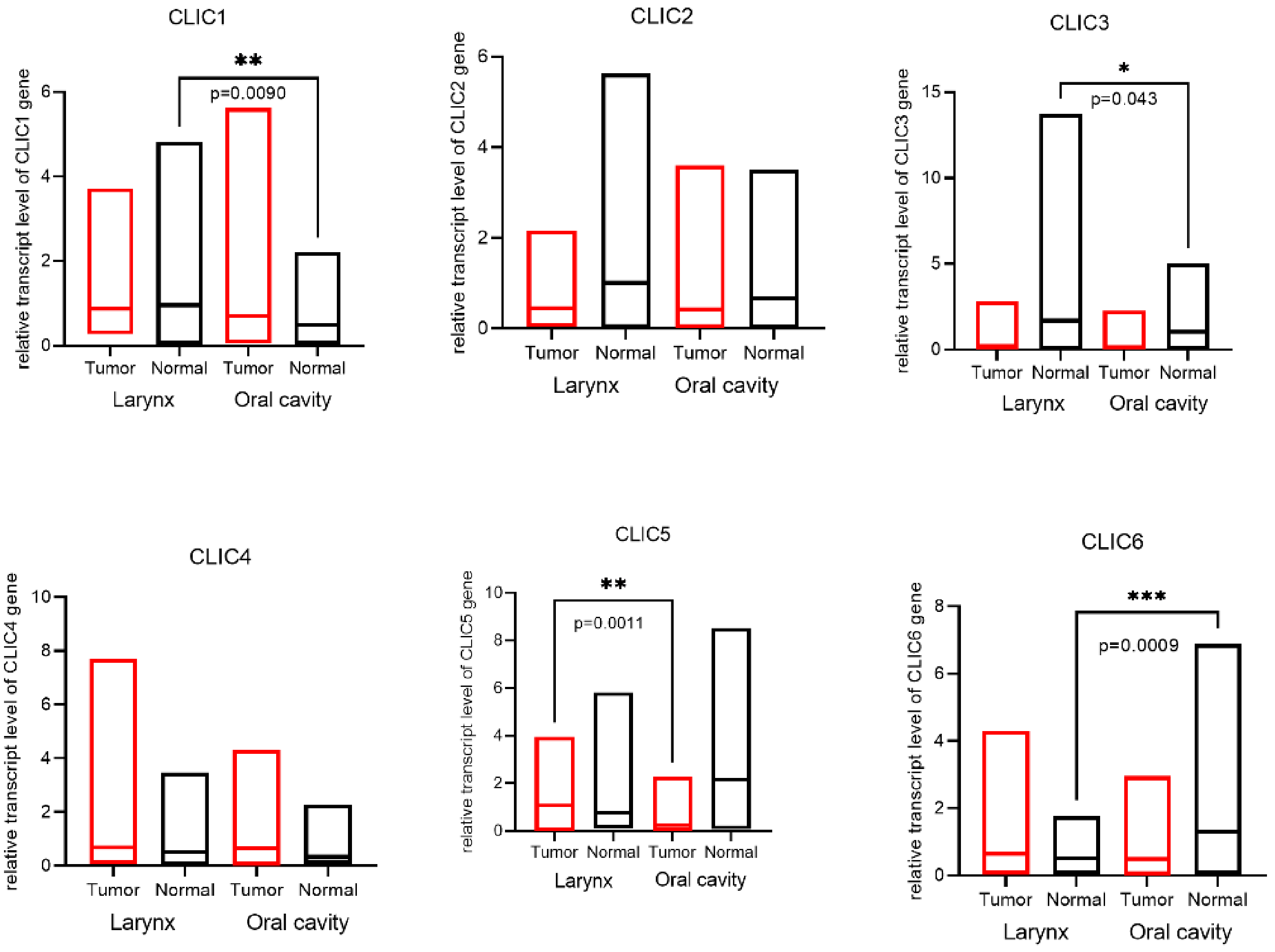

Supportive Information Figure 2. Spearman correlation matrix of *CLICs* mRNA expression in HNSCC tumors, presented as a heatmap showing Spearman's r values.

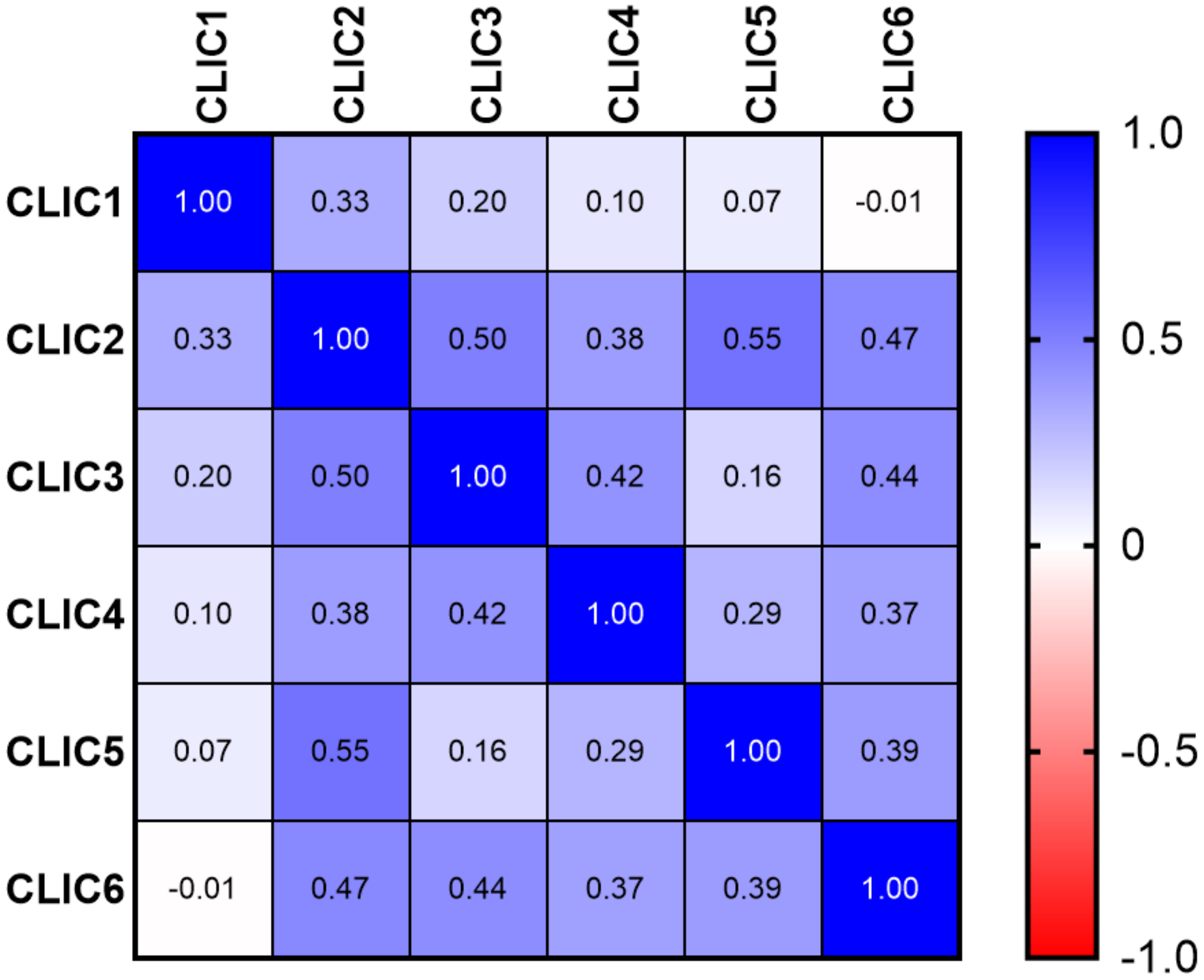

Supportive Information Figure 3. Spearman correlation matrix of CLICs protein expression in HNSCC tumors, presented as a heatmap showing Spearman's r values.

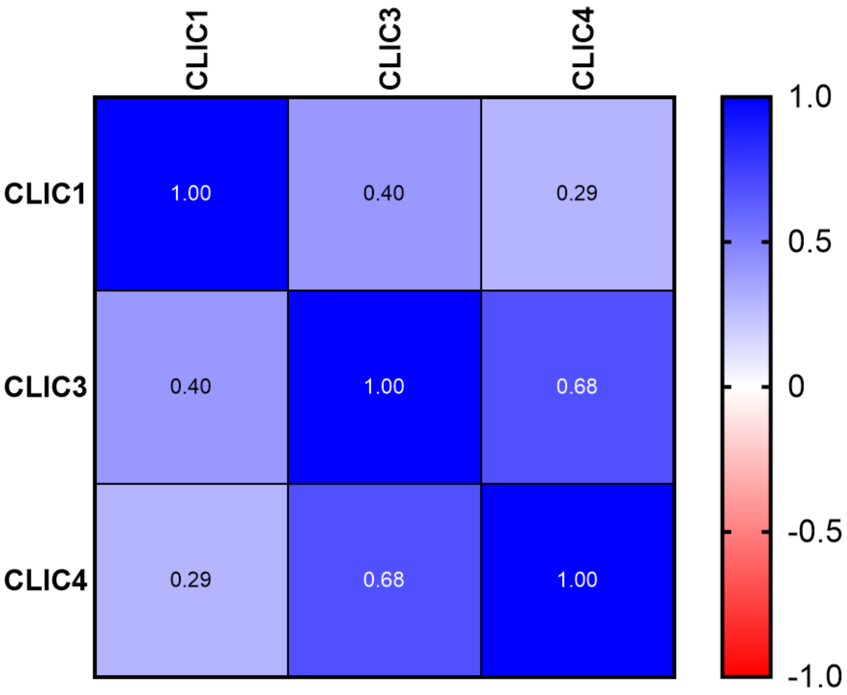

Supportive Information Figure 4. Spearman correlation matrix of CLICs protein expression in blood serum of HNSCC patients, presented as a heatmap showing Spearman's r values.

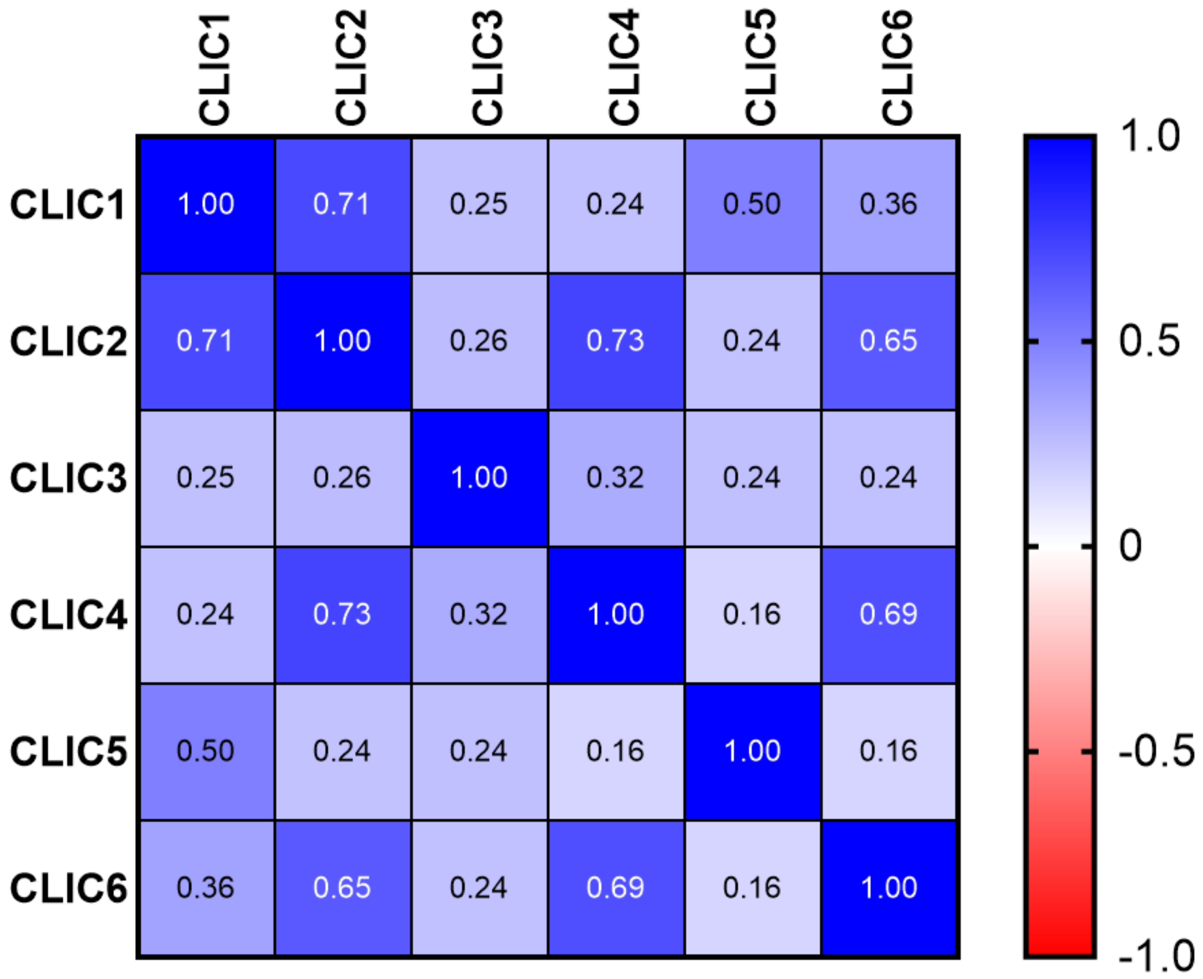

Supplement: S1 File — (PDF) [file pone.0333487.s001.pdf]
