# Supplementary material for: The pursuit of novel head and neck cancer biomarkers – tissue and blood expression of chloride intracellular channels family
Source: PLoS One. 2025 Oct 24;20(10):e0333487. doi: 10.1371/journal.pone.0333487 (PMC12551828; doi:10.1371/journal.pone.0333487)

S2.2. Original blots for the Figure 1B: CLIC1 and β-tubulin.

CLIC1

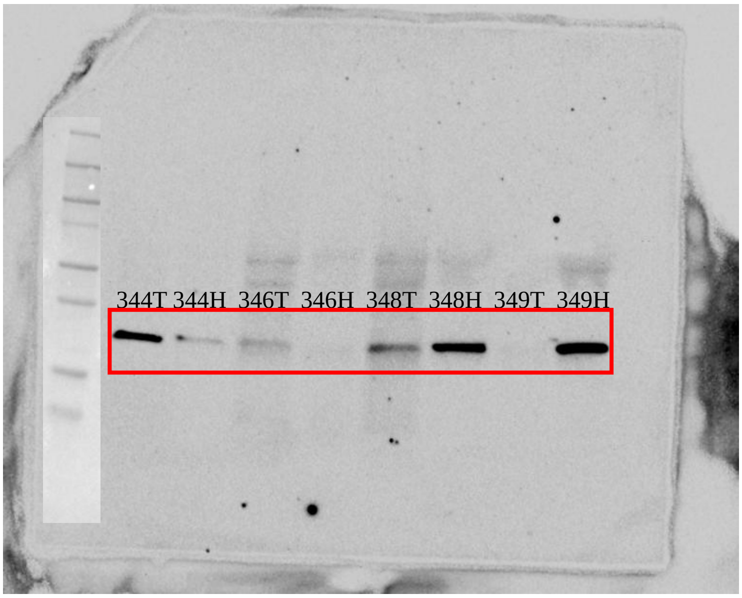

B-tubulin

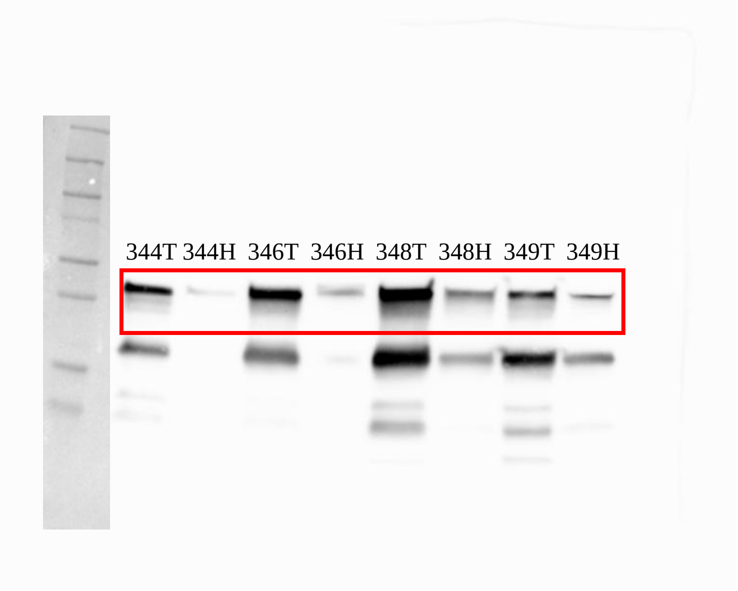

S2.2. Original blots for the Figure 1B: CLIC3 and β-tubulin.

CLIC3

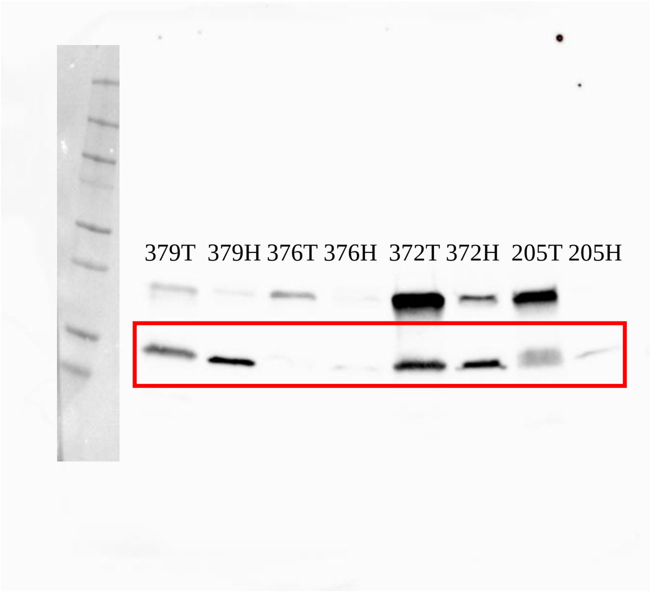

B-tubulin

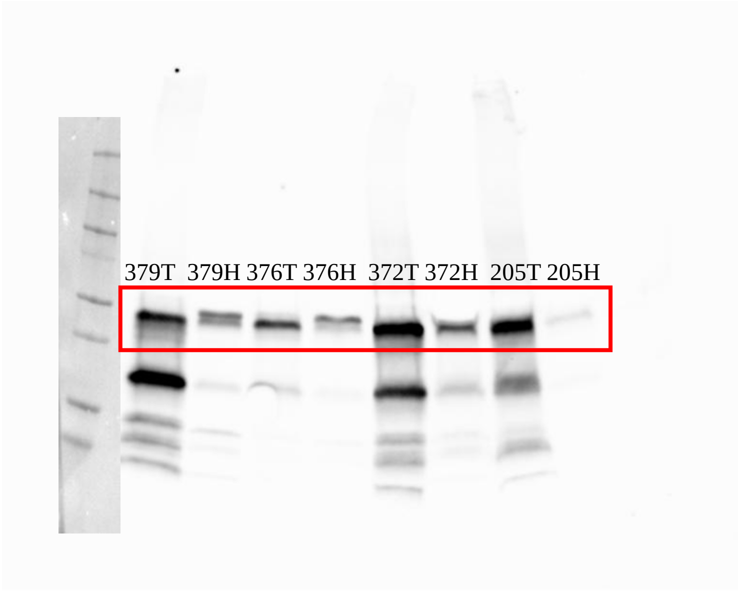

S2.3. Original blots for the Figure 1B: CLIC4 and β-tubulin.

CLIC4

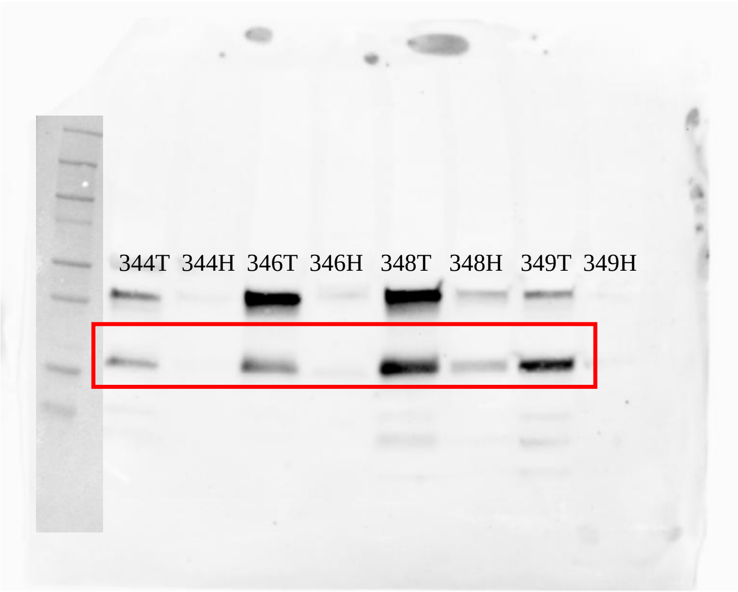

B-tubulin

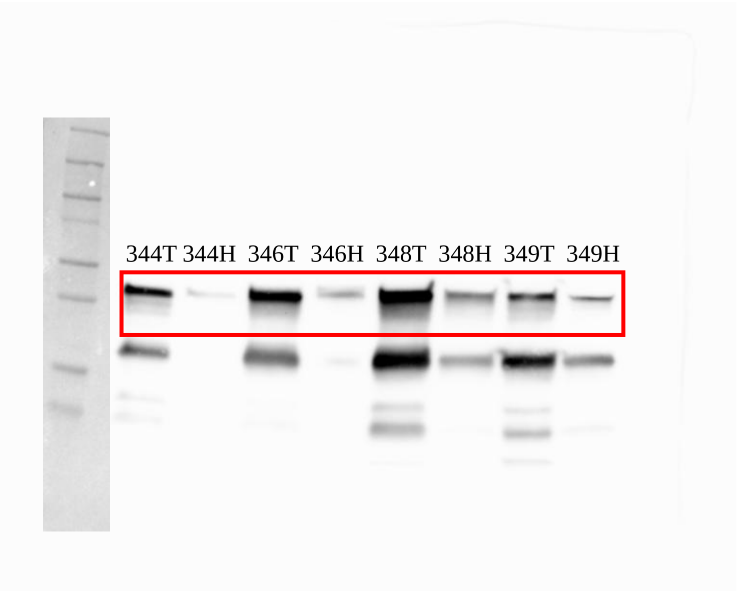

Supplement: S2 File — (PDF) [file pone.0333487.s002.pdf]
